# Prevalence of tuberculosis respiratory symptoms and associated factors in the indigenous populations of Paraguay (2012)

**DOI:** 10.1590/0074-02760160443

**Published:** 2017-07

**Authors:** Sarita Aguirre, Celia Martínez Cuellar, María Belén Herrero, Gustavo Chamorro Cortesi, Nilda Gimenez de Romero, Mirian Alvarez, Jose Ueleres Braga

**Affiliations:** 1 Ministry of Public Health and Social Welfare of Paraguay, Tuberculosis Control Program, Asunción, Paraguay; 2 Universidad Nacional de Asunción, Facultad de Ciencias Médicas, Asunción, Paraguay; 3 Facultad Latinoamericana de Ciencias Sociales, Department of International Relations, Buenos Aires, Argentina; 4 Ministry of Public Health and Social Welfare of Paraguay, Central Public Health Laboratory, Asunción, Paraguay; 5 Ministry of Public Health and Social Welfare of Paraguay, Department of Statistics of the Tuberculosis Control Program, Asunción, Paraguay; 6 Fundação Oswaldo Cruz-Fiocruz, Escola Nacional de Saúde Pública, Departamento de Epidemiologia e Métodos Quantitativos, Rio de Janeiro, RJ, Brasil; 7 Universidade do Estado do Rio de Janeiro, Instituto de Medicina Social, Departamento de Epidemiologia, Rio de Janeiro, RJ, Brasil

**Keywords:** pulmonary tuberculosis, population groups, cross-sectional studies, risk factors

## Abstract

**BACKGROUND:**

The prevalence of respiratory symptoms and confirmed tuberculosis (TB) among indigenous groups in Paraguay is unknown.

**METHODS:**

This study assessed the prevalence of respiratory symptoms, confirmed pulmonary TB, and associated socio-economic factors among indigenous Paraguayan populations. Indigenous persons residing in selected communities were included in the study. A total of 24,352 participants were interviewed at home between October and December 2012. Respiratory symptomatic individuals were defined as those with respiratory symptoms of TB. A hierarchical Poisson regression analysis was performed with four levels: individual characteristics, living conditions and environmental characteristics, source of food, and type of nutrition.

**FINDINGS:**

In this study, 1,383 participants had respiratory symptoms (5.7%), but only 10 had culture-confirmed TB (41/100,000 inhabitants). The small number of cases did not allow evaluation of the risk factors for TB. Age older than 37 years was associated with a two-fold increased risk of symptoms. Female sex; family history of TB; type of housing; home heating; a lack of hunting, fishing, or purchasing food; and a lack of vegetable consumption were also associated with the presence of symptoms. A lack of cereal consumption had a protective effect. Members of the Ayoreo or Manjui ethnic groups had a three-fold increased risk of symptoms.

**MAIN CONCLUSION:**

Individual characteristics, dietary habits, and belonging to specific ethnic groups were associated with respiratory symptoms.

Despite being a curable and preventable disease, tuberculosis (TB) remains a significant public health issue worldwide. The determinants of TB epidemiology include socioeconomic inequality, delayed diagnosis, and lack of social support for the care of sick individuals (Hargreaves et al. 2011). TB largely affects the vulnerable sections of the population, including indigenous populations (AFN 2009, Tollefson et al. 2013).

An approximate population of 370,000,000 indigenous persons worldwide comprises 5% of the global population (UN 2012). While there is evidence to suggest that the burden of TB increases considerably among the indigenous populations (Fanning 1999, Fitzgerald et al. 2000, Das et al. 2006, Barry & Konstantinos 2009, CDC 2010), the current global burden of TB remains unknown (Hoeppner & Marciniuk 2000, Culqui et al. 2010, Tollefson et al. 2013). The Stop TB Initiative highlighted the need to improve surveillance in indigenous villages in order to assess the burden of TB among these populations (AFN 2009).

Ten percent of the population in Latin America is indigenous (Culqui et al. 2010). The majority suffer discrimination and live in isolation and poverty. TB in these indigenous populations is associated with high poverty, migration, marginalisation, lack of territorial rootedness, environment destruction, and unmet basic needs (Culqui et al. 2010, Lopez et al. 2013). Poor living conditions increase the susceptibility to illness in these populations, and there are often language, geographical, and cultural barriers that lead to a delayed diagnosis of TB, which results in the delayed identification of transmission sources within the community and an increased risk of new cases (Culqui et al. 2010, Lopez et al. 2013, Tollefson et al. 2013).

According to the 2002 Census, there are 20 ethnic groups in Paraguay, which constitute 1.7% of the national population (DGEEC 2012). Based on the results of the 2012 Survey of Indigenous Households, approximately 112,800 persons belong to indigenous communities (DGEEC 2012).

TB is an endemic disease that constitutes a serious public health problem, primarily affecting the groups with the greatest levels of poverty in Paraguay (Lopez et al. 2013). Approximately 1,400 cases of pulmonary TB-positive smears are detected annually (24 cases per 100,000), while more than 200 cases are detected annually in the indigenous population, corresponding to 180 cases per 100,000 persons (http://vigisalud.gov.py/index.php/programa-nacional-de-control-de-la-tuberculosis/). The estimated incidence of respiratory symptoms (RS) and the prevalence of TB among the indigenous populations of Paraguay are not known; thus, this study primarily aims to fill this knowledge gap. The objective of this study was to assess the prevalence of RS and pulmonary TB among the indigenous population of Paraguay and to identify the major socio-economic factors associated with TB in this population.

## SUBJECTS AND METHODS

The study group consisted of the indigenous population residing habitually or permanently in indigenous communities nationwide. Any person living in a community for more than six months prior to the initial visit and who had not travelled outside the community for more than three of the previous six months was considered a habitual resident of the community. The inclusion criteria were as follows: persons belonging to any indigenous group in Paraguay, habitual residence within the selected communities, and voluntary verbal consent to participate in the survey.

The participants were interviewed in their homes by interviewers from the National Program for Tuberculosis Control (NPTC) of Paraguay, using a structured questionnaire to collect the study data. A maximum of three visits was made. The questionnaire applied in the survey collected data on demographic, economic, and housing characteristics, as well as information on personal and family history of TB, access to health services, and TB-related symptoms. The survey was organised in four sections: population characteristics, housing characteristics, prevalence of TB, and knowledge of TB.

A pilot test including 42 residents of the *Jukyty* indigenous community (not included in the study) was performed before the commencement of the survey. The participants in this study were provided a special form of informed consent to request their agreement to participate in the interview, which ensured ethical safeguards in accordance with the principles established in the Declaration of Helsinki.

Individuals with RS were examined, and two sputum samples were collected following fasting. Direct bacilloscopic examination was performed in the health services laboratories that attend this community, and culture examination was performed in the central laboratory after storage and delivery according to the standards of the National Tuberculosis Control Program of Paraguay.

Sputum samples were sent to the laboratories located closest to the community. The fieldwork lasted seven weeks (October-December, 2012). The sampling design was multistep, stratified with simple random sampling within the strata, with 94 communities selected from among 584 existing indigenous communities. The sample size was based on a prevalence of 0.5%, absolute error of 0.1%, finite population of 117,528 inhabitants, and design effect of 1.5. Thus, the calculated sample size was 24,266 indigenous persons.

Individuals with RS were defined as those reporting the presence of RS related to TB (a cough lasting three or more weeks, coughing up blood, chest pain, or pain when breathing or coughing). Pulmonary TB was defined as the presence of respiratory symptoms with a positive culture (Lowenstein-Jensen method).

A hierarchical Poisson regression analysis was performed to identify associated factors. The four levels (dimensions) of the analysis and their respective independent variables were: (*level 1*) Individual characteristics - age (< 38 years or > 38 years), sex (male/female), education level [Educación Escolar Básica (EEB) Level 1, 2, or ≥ level 3]; (*level 2*) living conditions and environmental characteristics - family history of TB (yes/no); type of housing; type of toilet (flush toilet/other); home heating type (none/some heating); access to a television (yes/no); ownership of horse, donkey, ox (yes/no); main cooking method (wood/other); (*level 3*) sourcing of food - personal garden (yes/no); hunting or fishing (yes/no); purchase of foods (yes/no); receipt of donations (yes/no); and (*level 4*) type of nutrition - vegetables (yes/no), cereals (yes/no), fruit (yes/no), meat (yes/no), dairy products (yes/no). An *“Ayoreo/Manjui Ethnicity”* variable was constructed, composed of the two ethnic groups with the highest prevalence of RS, and was included in the final multiple regression analysis.

The prevalence measures and their respective confidence intervals [95% CI (confidence interval)] were calculated. The prevalence ratio (PR) was calculated for the putative factors associated with RS. A simple Poisson regression was used to assess the individual effect of the independent variables on RS. Variables that were significant in the simple regression model (p < 0.20) were included in the respective multiple regression models. A multiple Poisson regression analysis was performed for each level, controlling for potential confounding variables. Only significant variables (p < 0.05) for each of the models were incorporated into the final regression model. The final model included variables with a significance level of p < 0.05. All statistical procedures were performed using Stata version 11.0.

## RESULTS

The interviewers visited 19 communities located across 13 departments. The survey included 24,352 people, 1,383 (5.7%) of whom had RS. Of these, 10 participants were culture-confirmed as having TB (41 per 100,000 inhabitants). The limited number of confirmed TB cases prevented the study of the associated factors.

Analysis of individual characteristics (*level 1*) revealed the highest frequency of RS among those of *Ayoreo* and *Manjui* ethnicities (Table I). Persons aged 38 to 95 years and divorcees had the highest RS prevalence. The RS prevalence also differed according to the work status of the household head. The RS prevalence was higher among female sex and individuals with a higher education status and without a visible BCG scar (Table I).

**TABLE I:** Individual characteristics of the survey population, stratified according to respiratory symptoms (level 1), Paraguay, 2012

	Respiratory symptomatic						p-value

	No		Yes		Total		
		
	n	(%)	n	(%)	N	(%)	
Ethnic group							
Aché	131	91,6	12	8,4	143	100	
Angaité	600	96,3	23	3,7	623	100	
Ava Guaraní	3936	95,9	170	4,1	4106	100	
Ayoreo	731	79,3	191	20,7	922	100	
Chamacoco Tomahoro	95	100	0	0	95	100	
Chamacoco Yvytoso	858	96,7	29	3,3	887	100	
Enxet	671	94,1	42	5,9	713	100	
Guana	22	95,7	1	4,3	23	100	
Guaraní Nandeva	445	96,1	18	3,9	463	100	
Guaraní occidental	944	97,6	23	2,4	967	100	
Lengua Enlhet Sur	2568	95,4	124	4,6	2692	100	
Maká	784	95,7	35	4,3	819	100	
Manjui	95	81,2	22	18,8	117	100	
Mbya	1941	93,4	138	6,6	2079	100	
Nivaclé	4347	93,7	291	6,3	4638	100	
Páî-Tavyterâ	2712	93,4	191	6,6	2903	100	
Sanapaná	280	97,2	8	2,8	288	100	
Toba Maskoy	366	95,8	16	4,2	382	100	
Toba-Qom	882	97,7	21	2,3	903	100	
Total	22408	94,3	1355	5,7	23763	100	< 0.001
Age (year groups)							
0-37 years	18490	95,4	881	4,6	19371	100	
38-95 years	4487	89,9	502	10,1	4989	100	
Total	22977	94,3	1383	5,7	23062	100	< 0.001
Sex							
Male	11835	95,1	611	4,9	12446	100	
Female	11080	93,5	771	6,5	11850	100	
Total	22915	94,3	1382	5,7	24296	100	< 0.001
Civil status							
Civil marriage	2471	92,3	205	7,7	2676	100	
Traditional wedding	2538	92,3	211	7,7	2749	100	
Separated	279	94,6	16	5,4	295	100	
Divorced	11	78,6	3	21,4	14	100	
Widower	379	84	72	16	451	100	
Single	13295	95,4	645	4,6	13940	100	
Cohabitation	3726	94,4	220	5,6	3946	100	
Total	22699	94,3	1372	5,7	24071	100	< 0.001
Education level							
Up to EEB 1° y 2° level	14088	94,8	768	5,2	14856	100	
EEB 3° level or above	8889	93,5	615	6,5	9504	100	
Total	22977	94,3	1383	5,7	24360	100	< 0.001
Household head work status							
Salaried	1.123	93,7	76	6,3	1.199	100	
Laborer	418	9,6	177	4,1	4.357	100	
Self employed	16.161	93,9	1.053	6,1	17.214	100	
None	1.449	95,1	75	4,9	1.524	100	
Total	19.151	78,8	1.381	5,7	24.294	100	< 0.001
Visible BCG scar							
Yes	10810	92,5	876	7,5	11686	100	
No	3398	91,3	324	8,7	3722	100	
Total	14208	92,2	1200	11,6	15408	100	0,017

Source: own elaboration.

Analysis of the living conditions and availability of goods and resources (*level 2*) revealed that RS were more frequent among participants with a family history of TB as well as among those living in makeshift housing, in houses roofed with wooden slats, or in houses with water tanks, no electricity, or with a deficient sanitary service and among those drinking untreated water, without electricity, or with deficient sanitary services. Significant differences were also observed among participants without a refrigerator, stove, phone, television, radio, or computer and without a means of transport or any heating system (Table II).

**TABLE II:** Living conditions and availability of goods and resources of the surveyed population, stratified according to respiratory symptoms (level 2), Paraguay, 2012

	Respiratory symptomatic						p-value

	No		Yes		Total		
		
	n	(%)	n	(%)	N	(%)	
Family history of TB							
No	20.705	94,7	1.149	5,3	21.854	100	
Yes	643	81,8	143	18,2	786	100	
Total	21.348	94,3	1.292	5,7	22.640	100	< 0.001
Type of housing							
House / Rancho	20.305	94,6	1148	5,4	21.453	100	
Makeshift shelter	2626	91,8	234	8,2	2.860	100	
Total	22.931	94,3	1382	5,7	24.313	100	< 0.001
Type of roofing							
Tile	970	94,3	59	5,7	1.029	100	
Straw	5.818	94,4	344	5,6	6.162	100	
Asbestos cement (Eternit)	1.926	95,4	93	4,6	2.019	100	
Zinc sheet	12.779	94	818	6	13.597	100	
Wooden tablet	81	93,1	6	6,9	87	100	
Reinforced concrete, earthenware	27	90	3	10	30	100	
Palm trunk	896	96,4	33	3,6	929	100	
Cardboard, rubber, packaging timber	426	94	27	6	453	100	
Total	22.923	94,3	1.383	5,7	24.306	100	0.021
Main source of water							
ESSAP	1.573	95,4	75	4,6	1.648	100	
Private Network (aguatería)	199	94,8	11	5,2	210	100	
Community Network (com.vecinal)	2.155	95,5	101	4,5	2.256	100	
Artesian well	1.574	94,2	97	5,8	1.671	100	
Common water well with curbstone with lid	1.207	94,4	72	5,6	1.279	100	
Common water well with curbstone capless	1.615	94,8	88	5,2	1.703	100	
Common water well without curbstone	857	94,1	54	5,9	911	100	
Cistern	7.041	93,1	524	6,9	7.565	100	
Tajamar, rising, river, stream	5.675	94,7	319	5,3	5.994	100	
Australian tank	72	96	3	4	75	100	
Water tank	924	96,5	34	3,5	958	100	
Total	22892	94,3	1.378	5,7	24.270	100	< 0.001
Treatment for drinking water							
Filtered	433	95,6	20	4,4	453	100	
Boiled	460	95	24	5	484	100	
Treated with sodium hypochlorite	1.652	95,5	78	4,5	1.730	100	
No treatment	20.332	94,2	1.260	5,8	21.592	100	
Total	22.877	94,3	1.382	5,7	24.259	100	0.066
Availability of electricity							
Yes (ANDE)	12.901	95,1	658	4,9	13.559	100	
Yes (Generated)	262	94,6	15	5,4	277	100	
None	9.752	93,2	710	6,8	10.462	100	
Total	22.915	94,3	1.383	5,7	24.298	100	< 0.001
Type of health service							
Bath water trawl (network/well)	676	96,4	25	3,6	701	100	
Other (Community, stream, river)	22301	94,3	1358	5,7	23659	100	
Total	22.977	94,3	1.383	5,7	24.360	100	0.014
Type of waste disposal							
Buried	1.142	95,6	53	4,4	1.195	100	
burned	14.223	94,6	808	5,4	15.031	100	
Thrown in water course	94	96,9	3	3,1	97	100	
Thrown outdoors	3.752	92,1	324	7,9	4.076	100	
Garbage collection	3.719	95,1	193	4,9	3.912	100	
Total	22.930	94,3	1.381	5,7	24.311	100	< 0.001
Type of home heating							
None	11650	93,5	809	6,5	12459	100	
Other	11327	95,2	574	4,8	11901	100	
Total	22977	94,3	1.383	5,7	24.360	100	<0.001
Cooking methods							
Firewood	19096	94	1.222	6	20.318	100	
Other	3881	96	161	4	4042	100	
Total	22915	94,3	1.380	5,7	24.360	100	< 0.001
Access to a refrigerator							
Yes	5.060	96	212	4	5.272	100	
No	15.492	93,5	1.082	6,5	16.574	100	
Total	20.552	94,1	1.294	5,9	21.846	100	< 0.001
Access to a kitchen							
Yes	4.080	95,8	181	4,2	4.261	100	
No	16.298	93,6	1.113	6,4	17.411	100	
Total	20.378	94	1.294	6	21.672	100	< 0.001
Access to a cellphone							
Yes	13.799	94,7	768	5,3	14.567	100	
No	7.953	93,5	551	6,5	8.504	100	
Total	21.752	94,3	1.319	5,7	23.071	100	< 0.001
Access to a TV							
Yes	9.114	95,6	416	4,4	9.530	100	
No	12.014	93,1	894	6,9	12.908	100	
Total	21.128	94,2	1.310	5,8	22.438	100	< 0.001
Access to a radio							
Yes	16.328	94,5	956	5,5	17.284	100	
No	5.472	93,8	364	6,2	5.836	100	
Total	21.800	94,3	1.320	5,7	23.120	100	0.044
Access to a computer or notebook							
Yes	458	96,4	17	3,6	475	100	
No	19.638	93,9	1.267	6,1	20.905	100	
Total	20.096	94	1.284	6	21.380	100	0.024
Access to a car							
Yes	434	97,3	12	2,7	446	100	
No	19.621	93,9	1.269	6,1	20.890	100	
Total	20.055	94	1.281	6	21.336	100	0.003
Access to a motorcycle							
Yes	9.441	95,1	482	4,9	9.923	100	
No	11.609	93,3	833	6,7	12.442	100	
Total	21.050	94,1	1.315	5,9	22.365	100	< 0.001
Access to a horse, donkey, or ox							
Yes	870	96,6	31	3,4	901	100	
No	19.251	93,9	1.250	6,1	20.501	100	
Total	20.121	94	1.281	6	21.402	100	0.001
Access to a bicycle							
Yes	7.366	94	471	6	7.837	100	
No	13.102	94,1	818	5,9	13.920	100	
Total	20.468	94,1	1.289	5,9	21.757	100	0.689

Source: own elaboration.

Analysis of the modes of obtaining food and diet (*levels 3* and *4*) revealed that individuals with their own gardens had fewer RS. Conversely, RS were more frequently observed among participants who did not eat vegetables, meat, dairy, or fruit as well as among those with higher consumption of grain (Table III).

**TABLE III:** Mode of obtaining food (level 3) and type of feeding (level 4), Paraguay, 2012

Characteristics	Respiratory symptomatic						p-value

	No		Yes		Total		
		
	n	(%)	n	(%)	N	(%)	
Own cultivation							
Yes	9.025	94,6	520	5,4	9545	100	
No	12.411	93,8	825	6,2	13236	100	
Total	21.436	94,1	1.345	5,9	22781	100	0.013
Hunting or fishing							
Yes	4.388	95,5	205	4,5	4593	100	
No	16.372	93,7	1.099	6,3	17471	100	
Total	20.760	94,1	1.304	5,9	22064	100	< 0.001
Purchasing food							
Yes	22.257	94,4	1.316	5,6	23573	100	
No	604	92,1	52	7,9	656	100	
Total	22.861	94,4	1.368	5,6	24229	100	0.010
Daily frequency of food consumption							
Once a day	2.007	92,7	159	7,3	2166	100	
Twice per day	5.513	94,1	345	5,9	5858	100	
Three times per day	14.992	94,7	845	5,3	15837	100	
Four times a day	352	94,1	22	5,9	374	100	
Five times a day	21	80,8	5	19,2	26	100	
More than 5 times	16	88,9	2	11,1	18	100	
Total	22.901	94,3	1.378	5,7	24279	100	< 0.001
Consumption of vegetables							
Yes	19.756	94,4	1.169	5,6	20925	100	
No	2.695	93,2	197	6,8	2892	100	
Total	22.451	94,3	1.366	5,7	23817	100	0.008
Consumption of green vegetables							
Yes	18.783	94,9	1.009	5,1	19792	100	
No	3.755	91,1	365	8,9	4120	100	
Total	22.538	94,3	1.374	1,5	23912	100	< 0.001
Consumption of cereals							
Yes	20.024	94,1	1.253	5,9	21277	100	
No	2.554	95,6	117	4,4	2671	100	
Total	22.578	94,3	1.370	5,7	23948	100	0.002
Consumption of fruit							
Yes	13849	94,4	817	5,6	14.666	100	
No	8041	93,8	534	6,2	8.575	100	
Total	21890	94,2	1.351	5,8	23.241	100	0.039
Consumption of meat							
Yes	19288	94,7	1.085	5,3	20.373	100	
No	3127	91,7	283	8,3	3.410	100	
Total	22415	94,2	1.368	5,8	23.783	100	< 0.001
Consumption of dairy products							
Yes	12819	95	670	5	13.489	100	
No	9109	93,1	670	6,9	9.779	100	
Total	21928	94,2	1.340	5,8	23.268	100	< 0.001
Receiving food donations							
Yes	1.847	95,9	79	4,1	1926	100	
No	18.662	93,9	1.213	6,1	19875	100	
Total	20.509	94,1	1.292	5,9	21801	100	< 0.001

Source: own elaboration.

Univariate analysis showed that TB symptoms were associated with age, sex, educational level and family history of TB. Symptoms were also associated with the type of housing; availability of sanitary services; and the lack of a home heating system, personal garden, consumption of donated food and hunting or fishing, as well as the purchase of food. Diets without vegetables, meat and dairy, as well as the means used for cooking, were also positively associated with the presence of symptoms. Finally, diets that included cereals or fruit were also risk factors for the development of symptoms (Table IV).

**TABLE IV:** Univariate and multivariate analysis of the characteristics associated with the prevalence of respiratory symptoms among the indigenous population of Paraguay, by level and hierarchical final model

Characteristics	(%)	Univariate analysis by level			Multivariate analysis by level			Final Multivariate analysis		
		
		PR	95% CI	p-value	PR	95% CI	p-value	PR	95% CI	p-value
LEVEL 1										
Age										
0 – 37 years	63,7									
≥38 years	36,3	2.21	2.09-2.63	< 0.001	2.16	1.93-2.41	<0.001	1.85	1.60-2.09	< 0.001
Sex										
Male	44,2									
Female	55,8	1.32	1.21-1.50	< 0.001	1.32	1.18-1.46	<0.001	1.33	1.19-1.50	< 0.001
Education level										
Until EEB level 1 and 2	55,5									
EEB level 3 or more	44,5	0.79	0.71-0.88	< 0.001	0.86	0.77-0.96	0.008	2.43	2.04-2.94	< 0.001
LEVEL 2										
Family history of TB										
No	88,9									
Yes	11,1	3.57	2.94-4.16	< 0.001	3.33	2.77-4.00	<0.001			
Type of housing										
House/Rancho	83,1									
Makeshift shelter	16,9	1.52	1.32-1.75	< 0.001	1.25	1.07-1.46	0.004	1.19	1.02-1.39	< 0.001
Type of health service										
Bath water trawl (network / well)	1,8									
Other (Community, stream, river)	97,2	1.60	1.08-2.39	0.018						
Availability of heating at home										
Yes	41,5									
No	58,5	1.34	1.20-1.49	<0.001	1. 39	1.24-1.57	<0.001	1.34	1.19-1.51	< 0.001
TV access at home										
Yes	31,8									
No	68,2	1.58	1.41-1.78	<0.001	1.38	1.21-1.58	<0.001	1.33	1.19-1.55	< 0.001
Use of a a horse, donkey, ox?										
Yes	2,4									
No	97,6	1.77	1.24-2.53	0.002	1.58	1.11-2.27	0.011			
Cooking method										
Firewood	88,4	1.50	1.28-1.77	< 0.001	1.29	1.07-1.55	0.006			
Other	11,6									
LEVEL 3										
Receiving food donations										
Yes	6,1									
No	93,9	1.48	1.18-1.86	0.001	1.43	1.12-1.81	0.004			
Own cultivation										
Yes	38,7									
No	61,3	1.14	1.02-1.27	0.016						
Hunting or fishing										
Yes	15,7									
No	84,3	1.40	1.21-1.63	< 0.001	1.36	1.16-1.59	<0.001	1.30	1.11-1.53	0.001
Purchasing food										
Yes	96,2									
No	3,8	1.41	1.07-1.87	0.015	1.59	1.19-2.12	0.001	1.46	1.10-1.93	0.009
LEVEL 4										
Consumption of green vegetables										
Yes	0,3									
No	99,7	1.73	1.54-1.95	< 0.001	1.63	1.42-1.86	<0.001	1.22	1.07-1.74	0.003
Consumption of cereals										
Yes	91,5									
No	8,5	0.74	0.61-0.89	0.003	0.69	0.57-0.84	<0.001	0.72	0.59-0.89	0.001
Consumption of fruit										
Yes	60,5									
No	39,5	1.11	1.01-1.24	0.045	0.82	0.72-0.94	0.004			
Consumption of meat										
Yes	79,3									
No	23,6	1.55	1.36-1.77	< 0.001	1.38	1.19-1.60	<0.001			
Consumption of dairy products										
Yes	50									
No	50	1.37	1.23-1.53	< 0.001	1.22	1.08-1.39	0.001			
Etnia Ayoreo/Manjui										
No	84,6									
Yes	15,4							3.25	2.75-3.85	< 0.001

CI: confidence interval. Source: own elaboration.

The lack of a heating system and the use of a wood-fired stove increased the risk for RS. The lack of access to a television, means of transport by animal and donated food were associated with an increased risk of RS (Table IV).

The final hierarchical analysis (Table IV) showed that age > 37 years increased the risk of symptoms by nearly two-fold. Female sex and family history of TB were also risk factors. The following factors were associated with an increased risk of RS: type of housing and home heating; not hunting or fishing; not purchasing food; and not consuming vegetables. Conversely, lack of grain consumption was a protective factor. Finally, belonging to the *Ayoreo* or *Manjui* ethnic groups increased the risk of symptoms by three-fold.

## DISCUSSION

The active identification of RS for the diagnosis of pulmonary TB is one of the most important tools from the perspective of public health. To our knowledge, this is the first study to estimate the prevalence of RS in an indigenous population in Paraguay and to address the burden of disease in this population.

The RS prevalence among indigenous communities in Paraguay in this study was 5.7% (1,383 cases). A total of 10 cases of TB were found. These results are comparable to those of large-scale studies conducted in other Latin American countries. Peru has reported an RS prevalence of 5% among the general population (Collazos et al. 2010). The estimated RS prevalence rates in two regions of Brazil are 5.7% and 4.8%, respectively (Freitas et al. 2011). Finally, Colombia reported an RS prevalence of 3.6% (García et al. 2004). The importance of these findings is that a significant proportion of respiratory symptomatic individuals may be pulmonary TB cases. In a study carried out in Vaupés (Colombia) to examine the prevalence of RS and TB and its associated factors, the prevalence of RS was 14.3%, with a 14-fold increase in the risk of TB in participants with RS (García et al. 2004). García et al. (2004) reported that the prevalence of RS among the indigenous population was almost twice that among non-indigenous persons. This observation is consistent with a study in Colombia, which reported that indigenous populations are much more likely to develop TB than non-indigenous populations in the area due to their generally deficient nutritional states and overcrowded conditions (Henao et al. 1999). Similarly, we also observed that the indigenous population in the current study was at risk of having symptoms. Furthermore, the results of our study show that individuals of *Ayoreo* or *Manjui* ethnicity had more than three-fold increased risk of having symptoms.

The findings of our study also suggest that the indigenous populations with RS suffer the worst deprivation, suggesting that RS prevalence is a multidimensional problem involving different factors related to individual characteristics, living conditions, social characteristics specific to the surrounding environment, type of nutrition and access to certain material goods. This is consistent with a study by Muniyandi et al. (2007), which observed a higher prevalence of RS and TB among populations exposed to greater poverty.

Living in makeshift homes, without a heating system and without a system for cooking are criteria that increase the risk of symptoms. Most previous studies did not assess the association between these housing characteristics and the prevalence of RS within the community. However, our results are consistent with the findings of a study conducted in two cities in Colombia by Daza Arana (2013), which concluded that living in poor housing with inadequate ventilation and in homes with roofs predominantly made of waste material were associated with an increased prevalence of RS (Moreno & Peña 2010). In addition, research conducted in Bucaramanga, Colombia (Nóbrega et al. 2010) found that RS was associated with households with no windows, with occupancies > 3 people per room, and a floor predominantly made of cement. This finding is consistent with the study by Krieger and Higgins, who reported that poor housing conditions were associated with health conditions, including the transmission of TB (Krieger & Higgins 2002).

Daza Arana (2013) concluded that the socioeconomic context influences the prevalence of RS and suggested that efficient methodologies of community-based research, based upon the specific characteristics of each territory, could be developed to further enhance the detection of RS (Daza Arana 2013). The author also found that a moderate level of food insecurity was associated with a greater risk of RS. Henao et al. (1999) also found that poor diet was associated with RS. The results of our study relating to nutrition conditions indicate that participants lacking vegetables in their diet had an increased risk of RS, while the lack of consumption of cereals proved to be a protective factor for the development of symptoms. Our study examined a low-income population; this finding may reflect the prevalent type of diet in this population. An example of this is the finding that the proportion of participants with RS was higher among those who did not practice hunting or fishing to obtain nutrition.

Freitas et al. (2011) found that RS was associated with lower socioeconomic status, as indicated by the lower income level in this population. The increased risk of RS was also associated with a lower level of education. The authors suggested that populations with low socioeconomic status have less access to health services (Freitas et al. 2011). Socioeconomic status and educational level may influence care seeking and attendance at health services. A study in Brazil found that barriers to access to health services in indigenous population influenced the presence of RS, as individuals were less likely to seek medical care (Nóbrega et al. 2010). In our study, the types of housing and home heating increased the risk of having symptoms. However, additional studies are necessary to investigate how socioeconomic factors contribute to increased incidence of RS.

The risk of RS among those with a family history of TB is indicative of disease transmission in this population. Moreover, after ethnic group, family history was the variable with the most significant impact on the increased risk of symptoms. Finally, in our study, female sex and older age were associated with a higher prevalence of RS. However, other studies have reported male sex to be a risk factor for RS. Our findings may be explained by the fact that the women in our study spent more time at home than did the men. In addition, indigenous women tend to minimise their symptoms and therefore delay seeking care (Thorson et al. 2004, WHO 2004). Women also act as the caregivers of the family, particularly for their children; because of their role in the household economy, women may consider the health of other household members before their own, as well as prioritising the family economy above any personal expenses. The finding of age as a risk factor is consistent with previous research (García et al. 2004). In this sense, the results of our study are consistent with other evidence suggesting that the risk of respiratory symptoms and developing TB increases with age.

Considering the small number of confirmed TB cases and the indicators of sensitivity and specificity, it is worth studying the factors. A systematic review of the sensitivity and specificity of questioning individuals regarding the presence of symptoms for the detection of bacteriologically confirmed active pulmonary TB in HIV-negative persons and persons with unknown HIV status considered eligible for TB screening revealed eight studies that provided data on ‘any TB symptom’ as a screen for symptomatic status. The number and duration of each symptom that qualified as a positive status differed across studies, ranging from four to eight symptoms. Cough, haemoptysis, fever, night sweats and weight loss were the most common (Tollefson et al. 2013). Thus, the results of our study are consistent with those of previous analyses, confirming that the presence of various symptoms in RS, particularly cough lasting three or more weeks, was the most frequent symptom.

This study has several limitations. In view of the small number of TB cases detected, it was not possible to examine the socioeconomic factors associated with TB. In addition, due to the complexity of the fieldwork and process of data collection in these communities, there was some loss of information and missing data. However, this does not imply a non-response bias in our study.

Our findings also trigger some reflections. Several variables across the different levels were significant in themselves. The fact that several factors individually and in context were associated with RS in the regression analysis shows that the prevalence of RS is socially determined and associated with poor living conditions. Further research is needed in order to better understand the association between the prevalence of RS, the underlying social and economic context, and the individual characteristics of participants, as well as to evaluate the relative contribution of each of these factors with the increased prevalence of RS among indigenous populations in Paraguay.

In conclusion, the findings of our study indicate that individual factors, dietary habits, and ethnic groups were associated with an increased prevalence of RS among indigenous populations in Paraguay. The detection of symptoms could enable the identification of targeted actions, optimisation of resources, and elaboration of long and short-term policies to reduce the prevalence of TB and contribute to disease control. Finally, the ethnic groups with higher prevalence are not explained by the current literature but offer a valuable finding for later investigations to elucidate why the RS differ in these indigenous groups.
